# Development and validation of a tumor marker-based model for the prediction of lung cancer: an analysis of a multicenter retrospective study in Shanghai, China

**DOI:** 10.3389/fonc.2024.1427170

**Published:** 2024-10-31

**Authors:** Sheng Hu, Qiang Guo, Jiayue Ye, Hongdan Ma, Manyu Zhang, Yunzhe Wang, Bingen Wan, Shengyu Qiu, Xinliang Liu, Guiping Luo, Wenxiong Zhang, Dongliang Yu, Jianjun Xu, Yiping Wei, Linxiang Zeng

**Affiliations:** ^1^ Department of Thoracic Surgery, The Second Affiliated Hospital of Nanchang University, Nanchang, China; ^2^ Department of Otolaryngology, The First Hospital of Nanchang, Nanchang, China; ^3^ Department of Medical Iconography, Xinfeng Maternal and Child Health Hospital, Ganzhou, China; ^4^ Department of Pulmonary and Critical Care Medicine, The Second Affiliated Hospital of Nanchang University, Nanchang, China

**Keywords:** tumor markers, lung cancer, nomogram, predictive models, development and validation

## Abstract

**Background:**

The incidence and mortality rates of cancer are the highest globally. Developing novel methodologies that precisely, safely, and economically differentiate between benign and malignant lung conditions holds immense clinical importance. This research seeks to construct a predictive model utilizing a combination of diverse biomarkers to effectively discriminate between benign and malignant lung diseases.

**Methods:**

This retrospective study included patients admitted to the two general hospitals in Shanghai from 2014 to 2015. This study was developed using five tumor markers: carcinoembryonic antigen (CEA), carbohydrate antigen 199 (CA199), cytokeratin fragment 21-1 (CA211), squamous cell carcinoma antigen (SCC), and neuron specific enolase (NSE). The entire sample was divided into two groups according to the hospital: 1033 cases were included in the development cohort and 300 cases in the validation cohort. Logistic regression analysis was used for univariate analysis to explore individual correlations between each selected clinical variable and lung cancer diagnostic outcome. Diagnostic prediction models were constructed and validated based on independent prognostic factors identified using multifactorial analysis. A nomogram was created using these tumor markers (age and sex were additionally included) and validated using the concordance index and calibration curves. Clinical prediction models were evaluated using decision curve analysis.

**Results:**

Fully adjusted multivariate analysis showed that the risk of lung cancer was 2.38 times higher in men than in women. CEA positivity was associated with an 13.41-fold increased risk in lung cancer. The area under the curve (AUC) values for the development cohort and validation cohort models were 0.907 and 0.954, respectively. In the established nomogram, the AUC for the receiver operating characteristic curve was 0.907 (95% CI, 0.889–0.925). The validation model confirmed the strong discriminative power of the nomogram (AUC = 0.954). The described calibration curves demonstrated good fit predictions and observation probabilities. In addition, decision curve analysis concluded that the newly established nomogram has important implications for clinical decision making.

**Conclusions:**

Combined prediction models based on CEA, CA199, CA211, SCC, and NSE biomarkers could significantly the differentiation between benign and malignant lung diseases, thus facilitating better clinical decision making.

## 1 Introduction

Lung cancer has the highest incidence and mortality rate in the world (1–5). Lung cancer screening is now commonly performed using low-dose computed tomography (LDCT), with or without other ancillary tests, such as sputum cytology. High-quality evidence suggests that LDCT screening significantly reduces lung cancer mortality and all-cause mortality in selected high-risk populations (6, 7). The size, density, shape, composition ratio, and CT signals are the primary factors used to make a clinical diagnosis of benign or malignant pulmonary nodules. On a single CT, it is challenging to discriminate benign from malignant disease because of the variety of lung nodules, and studies have shown that the proportion of benign lung nodules can increase to as much as 24% after surgical therapy (5, 8, 9). Currently, a range of screening techniques, including both noninvasive and invasive methods, have been proposed to predict the probability of malignancy in lung cancer detected by LDCT (4, 10–13). Each technique has benefits and drawbacks. To evaluate if it is a benign lesion, noninvasive techniques include follow-up positron emission tomography, LDCT, or magnetic resonance imaging for up to 2 years. For people with benign lesions, these noninvasive techniques typically lead to unnecessary radiation exposure, anxiety, surgery, and extra costs (14–17). A specific benign or malignant diagnosis can be made using a CT-guided percutaneous lung aspiration biopsy; however, it is intrusive, possibly dangerous, and occasionally malignant disease goes unnoticed (2, 18–22). Therefore, it is crucial for clinical practice to develop new methods for accurately, safely, and inexpensively identify individuals with benign and malignant lung disease (2, 6, 23).

Additionally, previous studies demonstrated that prediction models created using demographic information about participants and radiological properties of lung nodules on CT images can distinguish between benign and malignant lung disease (24, 25). For instance, Swensen et al. created the Mayo Clinic model with an area under curve (AUC) of 0.83 to identify malignant pulmonary nodules based on six independent variables (patient age, smoking history, cancer history, nodule diameter, upper lobe placement, and spines) (25). Even though these clinical/radiological feature-based models showed promise in distinguishing between benign and malignant lung illnesses, their diagnostic accuracies need to be increased (26).

In recent years, the search for biomarkers in body fluids has become an attractive approach, showing good progress (6, 27–31). For example, many antigens found in blood have been evaluated as potential biomarkers for lung cancer. The most studied biomarkers include cytokeratin fragment 21-1 (CYFRA 21-1), carcinoembryonic antigen (CEA), neuron-specific enolase (NSE), and squamous cell carcinoma antigen (SCC-Ag) (6). CA19-9 stands as a widely acknowledged circulating biomarker indicative of pancreatic cancer. Elevated serum CA199 levels, measured through the utilization of monoclonal antibodies, have been extensively employed as diagnostic or prognostic biomarkers for pancreatic cancer. Nevertheless, a plethora of studies posit a correlation between CA199 and lung cancer. It is noteworthy that glycoantigens 199 may manifest elevated levels in individuals afflicted with lung cancer. Furthermore, an escalated CA199 not only enhances the sensitivity and specificity of lung cancer diagnosis but also contributes to the precise prediction of intrapulmonary and distant metastases (32–35). Given the intricate nature of the tumor microenvironment and the phenomenon of clonal selection during the progression of lung cancer, reliance solely upon a single circulating biomarker or clinical/radiological factors may prove inadequate for the precise diagnosis of lung cancer. The aim of this study was to develop a predictive model based on the combination of multiple biomarkers to identify benign and malignant lung diseases. We combined CEA, carbohydrate antigen 199 (CA199), CYFRA211, SCC, and NSE in the modeling cohort and training cohort, respectively, to assess benign and malignant lung disease. The combination of biomarkers yielded an AUC of 0.907.

## 2 Materials and methods

### 2.1 Population and data sources

This is a secondary investigation involving data deposited within the Dryad public repository originating from a multicenter retrospective cohort study conducted in Shanghai (36). Patients admitted to the departments of oncology (Hospital A), thoracic surgery (Hospital B), and respiratory medicine (Hospital C) within the precincts of three prominent medical institutions in Shanghai, spanning the temporal expanse from January 2014 to December 2015, were encompassed within the antecedent retrospective analysis. The inclusion criteria for patients comprised a primary diagnosis of acute exacerbation of chronic obstructive pulmonary disease (COPD) and primary lung cancer (PLC) upon admission. Patients afflicted with COPD were diagnosed in accordance with the diagnostic parameters delineated in the Guidelines for the Diagnosis and Treatment of Chronic Obstructive Pulmonary Disease (2007 edition), a directive propounded by the Chinese Academy of Respiratory Sciences. As for patients with PLC, their inclusion was contingent upon meeting the stringent criteria for tumor lymph node metastasis (TNM) staging as per the International Union Against Cancer’s Lung Cancer standards, and these findings were duly corroborated through a combination of pathological examinations and imaging techniques. The meticulous scrutiny of all medical records was diligently undertaken by two seasoned physicians. Exclusion criteria were diligently applied to patients manifesting nonpulmonary conditions (e.g., surgical, cardiovascular, and cerebrovascular ailments), as well as those subjected to routine diagnoses with abbreviated hospital stays. The exclusion of cases from Hospital B in the subsequent analysis merits attention. This decision emanated from a strategic endeavor to independently formulate the modeling cohort and validation cohort across disparate medical facilities. The rationale behind this strategic planning is underscored by the fact that merely two out of the 102 patients from Hospital B received a diagnosis of lung cancer, a numerical inadequacy that precluded the execution of robust statistical analyses.

This study selected technically mature items and was developed using five tumor markers (TMs): CEA, CA199, CA211, SCC, and NSE. All sample collection was based on mature and clinically applied tumor biomarker testing techniques. Please indicate that the blood collection time is before treatment. All samples were quantitatively analyzed for markers using chemiluminescence or electroluminescence. These criteria were used to choose the participants. This study was a secondary analysis of a retrospective study, and the dataset was collected by Zhang et al. and is now available on the Dryad public database (https://doi.org/10.5061/dryad.nb3r0).The previousstudy protocol was approved by the Ethics Committee of Shanghai Xuhui Central Hospital. Because research using public databases does not involve information data and informed consent authorization issues of our institution, in addition to the fact that no personal privacy or identifiable information was involved in this study, the Second Affiliated Hospital of Nanchang University waived the ethical review of this study.

### 2.2 Cohort selection

**Figure 1:**
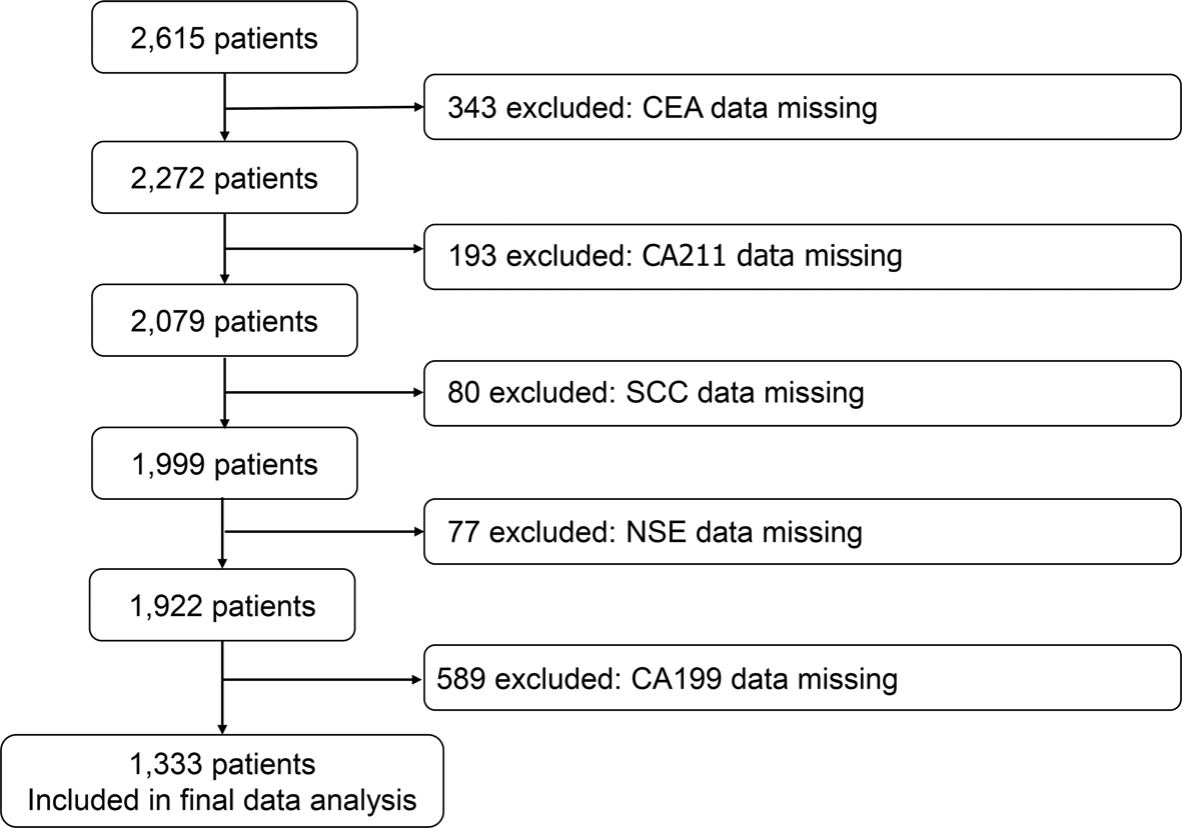
Participants’ screening flowchart.

We reviewed the registry for eligible cases and control individuals. We extracted case data from two of the hospitals with high numbers of cases, and details on the following variables were included in the case list: age, sex, year of diagnosis, hospital, and the levels of CEA, CA199, CA211, SCC, and NSE in serum. These candidate predictive model correlates were pre-determined based on analysis of peer-reviewed research literature that previously demonstrated their association with lung carcinogenesis or biological plausibility in lung cancer pathophysiology. The participants were divided into three groups according to age: < 70 years, ≥ 70 and < 80 years, and ≥ 80 years. We identified 2615 cases, excluding 343 patients with missing CEA data, 193 patients with missing CA211 data, 80 patients with missing SCC data, 77 patients with missing NSE data, and 589 patients with missing CA199 data. The model only contains participants who did not have any candidate predictors with missing data. 1333 cases were eventually included in the cohort (**Figure 1**).

### 2.3 Statistical analysis

The entire sample was divided into two groups according to hospital, 1033 cases were included in the development cohort (C hospital) and 300 cases in the validation cohort (A hospital). To investigate the individual relationships between each chosen clinical characteristic and lung cancer diagnostic results, logistic regression was employed for univariate analysis. Diagnostic prediction models were then developed, and the models were constructed and validated based on independent prognostic factors identified by multifactorial analysis. The consistency statistic (C-statistic), which is displayed as the area under the subject’s receiver operating characteristic (ROC) curve, was used to determine the main result, which represented the discriminatory accuracy of the model in predicting lung cancer. AUC areas over 0.7 indicated effective model discrimination. The Hosmer–Lemeshow test was used to assess model discrimination and 10 fold cross-validation was used for internal validation. Calibration curves were plotted to assess the degree of fit between the column line plot predictions and the actual situation. Empower (R) (www.empowerstats.com, X&Y solutions, Inc. Boston, MA, USA) and R version 3.6.3 (http://www.R-project.org) were used for all analyses. Empower Stats is a statistical software based on the R language for data analysis. The software has powerful data processing functions, as well as comprehensive analysis functions. The agreed cut off for statistical significance was P < 0.05.

## 3 Results

### 3.1 Baseline characteristics of the study participants

A total of 1333 participants were included in the study cohort, of which 1033 (77.49%) were in the development cohort and 300 (22.51%) were in the validation cohort. The proportions of women and men were 28.66% and 71.34%, respectively. Age less than 70 years old, between 70 and 80 years old, and more than 80 years old accounted for 34.36%, 23.71%, and 41.94%, of the participants, respectively. 70.44% of the participants were positive for CEA, while the remaining 29.56% were negative. Overall, participants in the development and validation cohorts were comparable in terms of demographics and tumor marker characteristics (**Table 1**).

**Table 1:** Demographic and clinical characteristics of patients in the development and validation cohorts.

Variable	C hospital Development cohort	A hospital Validation cort	Total	P-value
N	1033(22.51%)	300(22.51%)	1333	
Age	74.68 ± 11.95	77.24 ± 12.50	75.26 ± 12.12	0.001
Disease				<0.001
Benign lung disease	479 (46.37%)	235 (78.33%)	714 (53.56%)	
Lung cancer	554 (53.63%)	65 (21.67%)	619 (46.44%)	
Year of diagnosis				0.128
2014	629 (60.89%)	168 (56.00%)	797 (59.79%)	
2015	404 (39.11%)	132 (44.00%)	536 (40.21%)	
Gender				0.148
Female	306 (29.62%)	76 (25.33%)	382 (28.66%)	
Male	727 (70.38%)	224 (74.67%)	951 (71.34%)	
Age_categorical				0.017
≤70	370 (35.82%)	88 (29.33%)	458 (34.36%)	
≥70,<80	251 (24.30%)	65 (21.67%)	316 (23.71%)	
≥80	412 (39.88%)	147 (49.00%)	559 (41.94%)	
CEA				<0.001
Negative	698 (67.57%)	241 (80.33%)	939 (70.44%)	
Positive	335 (32.43%)	59 (19.67%)	394 (29.56%)	
CA199				0.35
Negative	829 (80.25%)	248 (82.67%)	1077 (80.80%)	
Positive	204 (19.75%)	52 (17.33%)	256 (19.20%)	
CA211				<0.001
Negative	520 (50.34%)	280 (93.33%)	800 (60.02%)	
Positive	513 (49.66%)	20 (6.67%)	533 (39.98%)	
SCC				0.192
Negative	618 (59.83%)	192 (64.00%)	810 (60.77%)	
Positive	415 (40.17%)	108 (36.00%)	523 (39.23%)	
NSE				<0.001
Negative	752 (72.80%)	290 (96.67%)	1042 (78.17%)	
Positive	281 (27.20%)	10 (3.33%)	291 (21.83%)	

Continuous variables were presented as mean ± SD; Categorical variables were presented as n (%). CEA, carcinoembryonic antigen; CA199, carbohydrate antigen 199; CA211, cytokeratin fragment 21-1; SCC, squamous cell carcinoma antigen; NSE, neuron-specific enolase. The p-values are comparing differences between hospital C and A.

### 3.2 Univariate and multivariate analyses of predicted factors in the development cohort

To demonstrate potential correlations between each individual predictor and the results, we first carried out univariate analysis (**Table 2**). We selected the seven factors in **Table 2**, but not year of diagnosis, as predictors for the model: Sex, age, CEA, CA199, CA211, SCC, and NSE. All seven factors chosen as potential predictors had a strong correlation with the univariate analysis’s outcome variables. The final model considered all seven factors.

**Table 2:** Univariate and multivariate analyses of factors associated with diagnosis in the development cohort.

Variable	Statistics N (%)	Univariate analysis		Multivariate analysis					
				No-adjusted		Model I		Model II	
		HR (95% CI)	*P* value	HR (95% CI)	*P* value	HR (95% CI)	*P* value	HR (95% CI)	*P* value
Year of diagnosis									
2014	629 (60.89%)	1		–	–	–	–	–	–
2015	404 (39.11%)	1.42 (1.10, 1.83)	0.0065	–	–	–	–	–	–
Gender									
Female	306 (29.62%)	1		1		1		1	
Male	727 (70.38%)	0.42 (0.32, 0.56)	<0.0001	0.42 (0.32, 0.56)	<0.0001	0.35 (0.25, 0.48)	<0.0001	0.40 (0.27, 0.60)	<0.0001
Age_categorical									
≤70	370 (35.82%)	1		1		1		1	
≥70,<80	251 (24.30%)	0.28 (0.20, 0.41)	<0.0001	0.28 (0.20, 0.41)	<0.0001	0.24 (0.17, 0.35)	<0.0001	0.17 (0.10, 0.27)	<0.0001
≥80	412 (39.88%)	0.08 (0.05, 0.11)	<0.0001	0.08 (0.05, 0.11)	<0.0001	0.07 (0.05, 0.10)	<0.0001	0.04 (0.03, 0.07)	<0.0001
CEA									
Negative	698 (67.57%)	1		1		1		1	
Positive	335 (32.43%)	13.41 (9.25, 19.44)	<0.0001	13.41 (9.25, 19.44)	<0.0001	12.12 (8.02, 18.32)	<0.0001	7.73 (4.82, 12.39)	<0.0001
CA199									
Negative	829 (80.25%)	1		1		1		1	
Positive	204 (19.75%)	4.62 (3.18, 6.69)	<0.0001	4.62 (3.18, 6.69)	<0.0001	5.21 (3.41, 7.96)	<0.0001	1.96 (1.11, 3.47)	0.021
CA211									
Negative	520 (50.34%)	1		1		1		1	
Positive	513 (49.66%)	3.38 (2.62, 4.37)	<0.0001	3.38 (2.62, 4.37)	<0.0001	6.79 (4.79, 9.63)	<0.0001	4.20 (2.77, 6.38)	<0.0001
SCC									
Negative	618 (59.83%)	1		1		1		1	
Positive	415 (40.17%)	0.60 (0.47, 0.77)	<0.0001	0.60 (0.47, 0.77)	<0.0001	0.93 (0.69, 1.25)	0.6177	0.44 (0.30, 0.65)	<0.0001
NSE									
Negative	752 (72.80%)	1		1		1		1	
Positive	281 (27.20%)	4.89 (3.54, 6.75)	<0.0001	4.89 (3.54, 6.75)	<0.0001	6.69 (4.55, 9.84)	<0.0001	5.31 (3.35, 8.42)	<0.0001

Non-adjusted model adjusted for: None. Model I model adjusted for: Age_categorical; Year of diagnosis (except the Stratification factor itself). Model II adjusted for: Year of diagnosis; Age_categorical; CEA; CA199; CA211; SCC; NSE (except the Stratification factor itself). CEA, carcinoembryonic antigen; CA199, carbohydrate antigen 199; CA211, cytokeratin fragment 21-1; SCC, squamous cell carcinoma antigen; NSE, neuron-specific enolase.

Based on independent prognostic indicators discovered through multifactorial analysis, the prediction model was created and verified (**Table 2**). In the fully adjusted model, the risk of lung cancer was found to be 2.38 times higher in men than in women. Lung cancer risk increased by 13.41-fold in people who tested positive for CEA. Patients who were positive for CA199, CA211, and NSE were all associated with a higher risk of lung cancer, at 4.62, 3.38, and 4.89 times higher than in patients who were negative for these factors, respectively. In contrast, SCC negativity was associated with a lower risk of lung cancer development. The adjusted variables are detailed in **Table 2**.

### 3.3 Model development and validation

**Figure 2:**
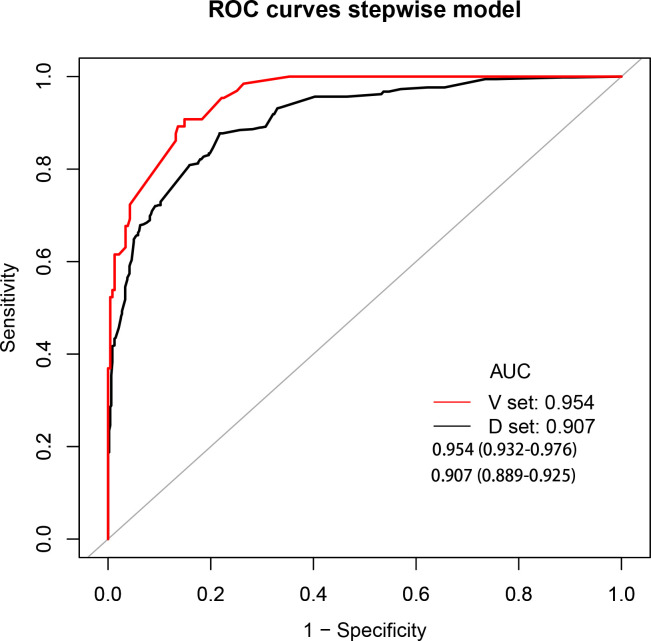
Receiver operating characteristic curve analyses. AUC, area under the curve; black curve, development cohort; red curve, validation cohort.

**Figure 3:**
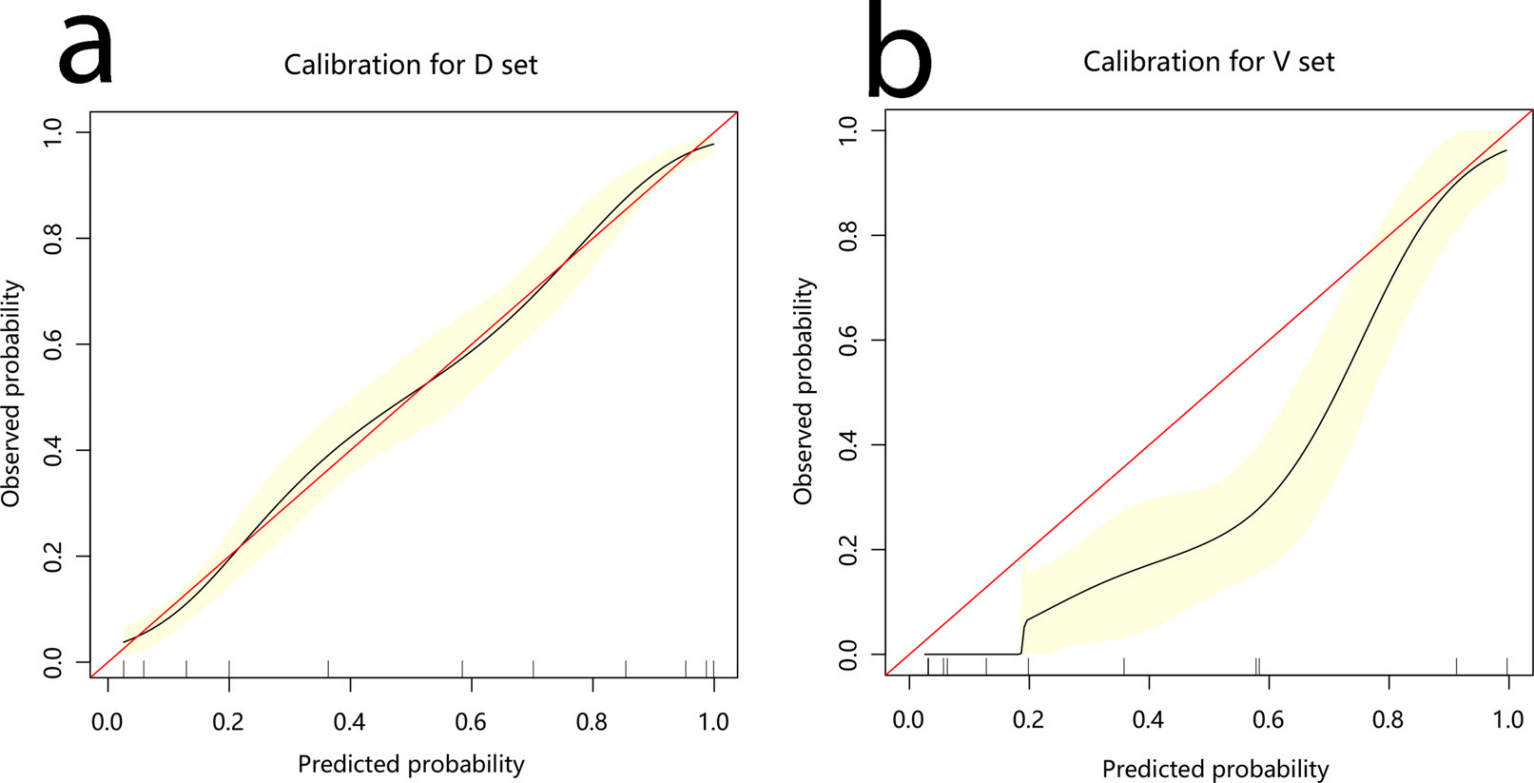
Calibration curve for the nomogram. **(A)** development cohort **(B)** validation cohort.

The development cohort comprised 1,033 participants. This included 554 cases and 479 controls (**Table 1**). The model’s performance measures are shown in **Figure 2**, revealing that cases and controls might be distinguished well from one another (AUC = 0.907, 95% confidence interval (CI): 0.889¬–0.925). The specificity, sensitivity, and accuracy were 0.8184, 0.8339, and 0.827 (95% CI: 0.802¬–0.849), respectively. In addition, the calibration plots showed that the model was well adapted (**Figure 3**).

Among the 300 patients included in the external validation cohort, 236 were annotated as benign lung disease and 65 were annotated as lung cancer. The AUC, specificity, sensitivity, and accuracy of the validation cohort model were 0.954, 0.8383, 0.8923, and 0.85 (95%CI: 0.804–0.888), respectively (**Figure 2**).

**Figure 4:**
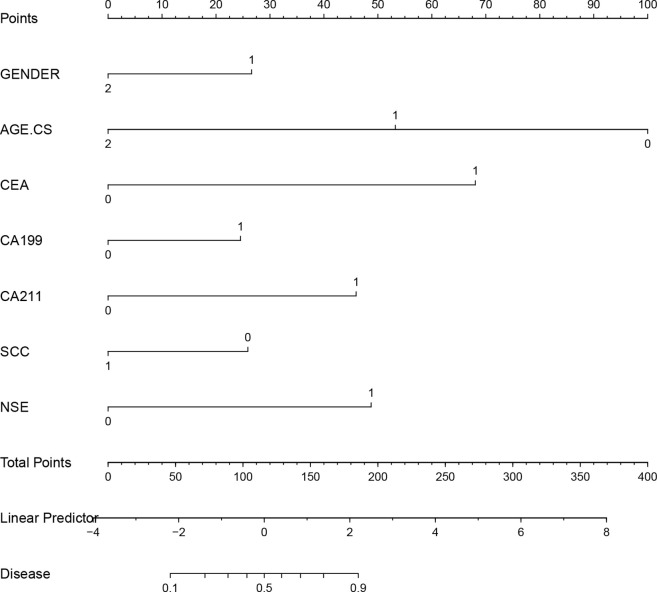
Nomogram predicting the probability of lung cancer in the development cohort.

Based on a multifactorial analysis of independent prognostic factors, we created a nomogram to predict the risk of lung cancer occurrence, and the probability of lung cancer occurrence in patients can be easily obtained from the nomogram by summing each selected variable (**Figure 4**).

### 3.4 Clinical use

**Figure 5:**
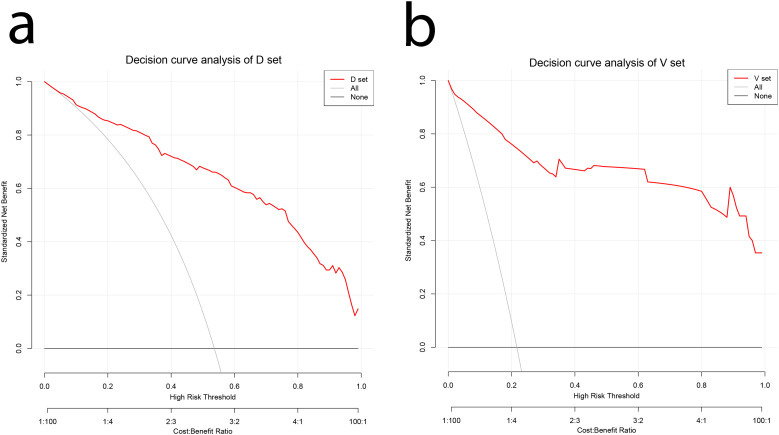
DCA curves for validating the net income of the nomogram. **(A)** Development cohort **(B)** Validation cohort. DCA, decision curve analysis.

**Figure 6:**
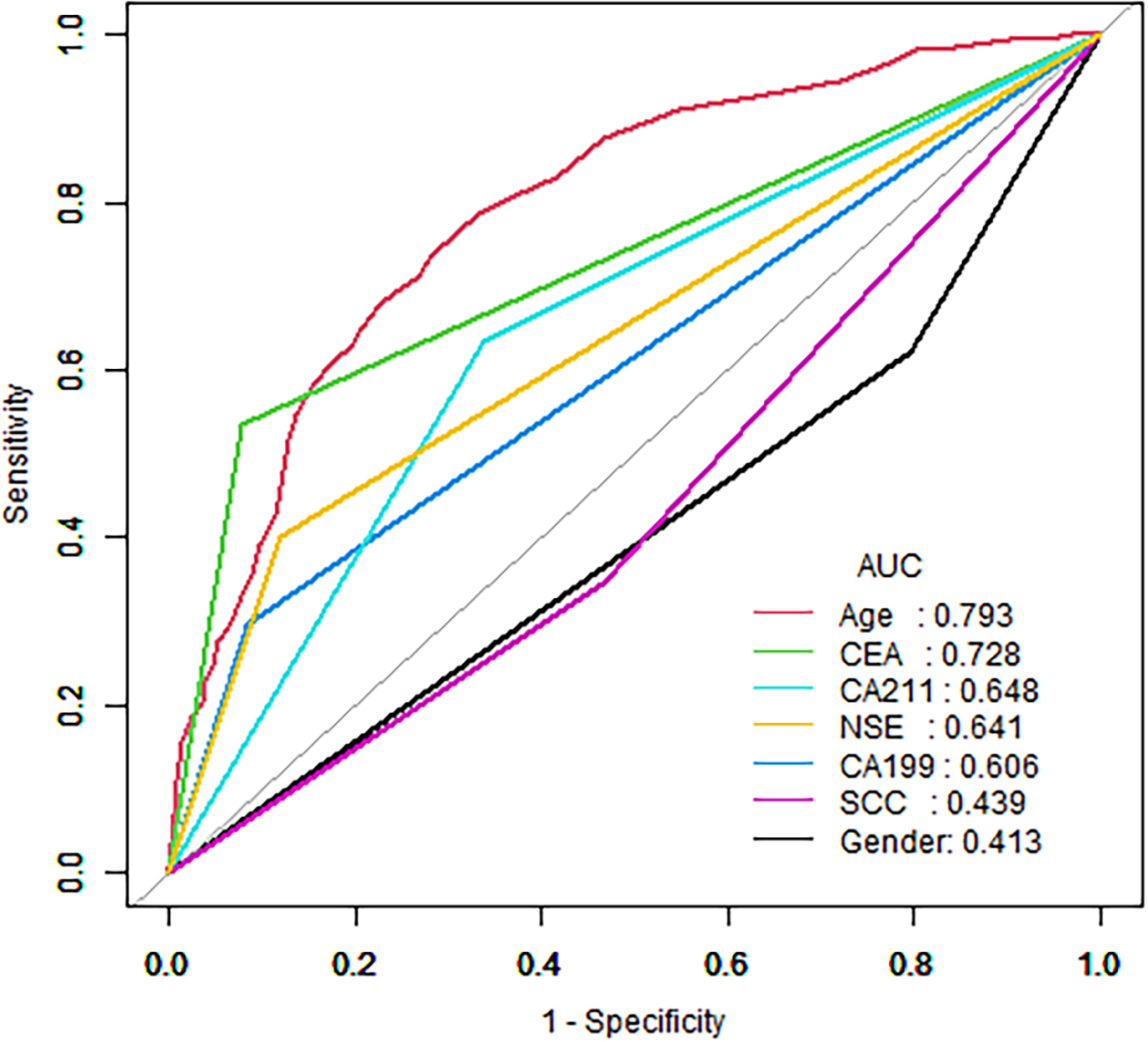
ROC curves and AUC values of individual protein biomarkers.

The decision curve analysis (DCA) of the nomogram and clinical prediction model are shown in **Figure 5**. **Figure 6** shows the ROC curves and AUC values for individual protein biomarkers. The comprehensive model provided a better net benefit in diagnosing patients with lung cancer, especially in the absence of available alternative predictive models.

## 4 Discussion

Our results suggested that a diagnostic model based on combined CEA, CA199, CA211, SCC, and NSE biomarkers could predict lung cancer more effectively. The AUC of the model for the development cohort was 0.907. the AUC or the validation cohort as 0.954. The results suggested that the prediction model based on the combination of CEA, CA199, CA211, SCC and NSE biomarkers could significantly improve the prediction of benign and malignant lung diseases, with sensitivities and specificities of 0.899 and 0.839, respectively. This combined biomarker prediction model has significant improvement in differentiating benign and malignant lung diseases and provides strong support for early diagnosis and differentiation of lung cancer. Compared with the traditional prediction model, this model has better accuracy, differentiation power and comprehensiveness. With the deepening research on lung cancer biomarkers and diagnostic models, this multi-indicator combination is expected to become an important tool for early screening and diagnosis of lung cancer in the future, and is expected to achieve wider application in clinical practice.

Wang et al. discussed biomarkers for lung cancer immunotherapy, including PD-L1 expression, tumor infiltrating lymphocytes (TILs), and tumor mutation burden (TMB), which can be used to select patients for immune checkpoint inhibitor (ICB) treatment. In addition, circulating tumor DNA (ctDNA) analysis and gut microbiota may also be used to predict ICB response (37).

Numerous lung nodules have been discovered as a result of the widespread use of LDCT in early lung cancer screening; however, there is an issue with overdiagnosis and overtreatment (7, 9, 38, 39). Additionally, prior research has demonstrated that prediction models can distinguish between benign and malignant lung nodules based on the demographics of participants and the radiological properties of lung nodules on CT scans (40). For instance, the Mayo Clinic model (25). Another predictive model based on age, smoking history, nodule diameter, and smoking cessation yielded an AUC of 0.78 (27). More recently, McWilliams et al. also developed two similar prediction models with AUCs of 0.89–0.91 (41). Although these models based on clinical/radiological features are promising to identify lung cancer, their diagnostic accuracy requires improvement. Considering the complex tumor microenvironment and clonal selection in the development of lung cancer, the use of circulating biomarkers alone or clinical/radiological factors alone might be insufficiently accurate to diagnose lung cancer (9). We found that predictive models based on combined lung cancer biomarkers can discriminate well between benign and malignant lung diseases, and the AUC values of the predictive models were higher than those of the Mayo Clinic model or the biomarker groups used alone.

In addition, the study subjects were from different hospitals and might represent a population from several distinct centers. The test methods and data generation processes differed between hospitals; however, the models built separately yielded better and closer AUCs, suggesting that our combined lung cancer biomarker diagnostic model might be applicable in multiple centers. We will also carry out a sizable population-based LDCT screening experiment to confirm how well the predictive algorithm distinguishes between benign and malignant nodules. Our study does, however, have certain drawbacks. The first is the small sample size, with only two medical centers included. Additionally, the original database’s missing data prevented us from undertaking further stratified analysis to produce more precise results because there was no information on staging or pathology type.

In clinical practice, it is crucial to distinguish between malignant nodules and non-malignant nodules. The existing tools provide important basis for doctors’ diagnosis. The Brock and Mayo risk models play an important role in this regard. The Brock risk model may evaluate the malignancy risk of nodules by comprehensively analyzing multiple clinical indicators, such as nodule size, morphology, patient age, gender, family history, and other factors. The Mayo Risk Model is also a tool that has been extensively validated through clinical practice. It may consider different combinations of factors, such as blood test indicators, imaging features, and patient symptom manifestations. The application of these risk models enables doctors to assess malignant risks more scientifically and objectively when facing patients with nodules, thereby formulating more reasonable diagnosis and treatment plans. This article aims to establish a simpler and more accessible diagnostic model using more objective and common levels of tumor markers in the blood (42, 43).

In conclusion, using a combination of CEA, CA211, NSE, SCC, and CA199 lung cancer biomarkers, we created a straightforward predictive model that could distinguish between benign and malignant lung disease after LDCT. Future application of the predictive model might result in cost savings and avoidance of invasive diagnostic procedures in people with benign lung disease, while enabling early treatment of patients with lung cancer. Lung cancer can be more accurately diagnosed by combining the prediction model and LDCT. However, prospective studies of the predictive models for lung cancer in the context of broad population-based LDCT screening are warranted.

## 5 Conclusions

Combined prediction models based on CEA, CA199, CA211, SCC, and NSE biomarkers could significantly improve the prediction of benign and malignant lung diseases, thus facilitating better clinical decision making.

## Data Availability

The original contributions presented in the study are included in the article/**Supplementary Material**. Further inquiries can be directed to the corresponding authors.
