# Supplementary material for: Development and validation of a tumor marker-based model for the prediction of lung cancer: an analysis of a multicenter retrospective study in Shanghai, China
Source: Front Oncol. 2024 Oct 31;14:1427170. doi: 10.3389/fonc.2024.1427170 (PMC11562644; doi:10.3389/fonc.2024.1427170)
Supplement: Supplementary file 1 [file Table1.docx]

|  | Sensitivity | Specificity |
| --- | --- | --- |
| Gender | 0.754 | 0.255 |
| Age | 0.754 | 0.834 |
| CEA | 0.615 | 0.919 |
| CA199 | 0.646 | 0.957 |
| CA211 | 0.200 | 0.970 |
| SCC | 0.246 | 0.609 |
| NSE | 0.046 | 0.970 |

Supplementary Table 1. The sensitivity and specificity of individual protein biomarkers.
