# Supplementary material for: Development and validation of a tumor marker-based model for the prediction of lung cancer: an analysis of a multicenter retrospective study in Shanghai, China
Source: Front Oncol. 2024 Oct 31;14:1427170. doi: 10.3389/fonc.2024.1427170 (PMC11562644; doi:10.3389/fonc.2024.1427170)
Supplement: Supplementary file 2 [file Table2.docx]

Supplementary Table 2. Univariate analysis of various protein biomarkers and age.

| Age | ≤70 | ≥70,＜80 | ≥80 |
| --- | --- | --- | --- |
| N(%) | 458 (34.36%) | 316 (23.71%) | 559 (41.94%) |
| CEA | 1.0 | 0.61 (0.45, 0.82) 0.0012 | 0.30 (0.22, 0.40) <0.0001 |
| CA199 | 1.0 | 0.85 (0.61, 1.20) 0.3613 | 0.42 (0.30, 0.58) <0.0001 |
| CA211 | 1.0 | 1.06 (0.79, 1.42) 0.6815 | 0.91 (0.70, 1.17) 0.4495 |
| SCC | 1.0 | 1.38 (1.02, 1.86) 0.0379 | 1.98 (1.53, 2.57) <0.0001 |
| NSE | 1.0 | 1.04 (0.75, 1.45) 0.8152 | 0.63 (0.46, 0.85) 0.0026 |

Data in the table: β (95% CI) P value/OR (95% CI) P value

Result variable:CEA; CA199; CA211; SCC; NSE

Exposed variable: Age
